# Supplementary material for: Lycium Barbarum Polysaccharide-Iron (III) Chelate as Peroxidase Mimics for Total Antioxidant Capacity Assay of Fruit and Vegetable Food
Source: Foods. 2021 Nov 14;10(11):2800. doi: 10.3390/foods10112800 (PMC8623380; doi:10.3390/foods10112800)
Supplement: Supplementary file 1 [file foods-10-02800-s001.zip › foods-1454240-supplementary.pdf]

1. Preparation of material *Lycium barbarum* polysaccharide (LBP)

1.1. Purification of crude LBP

5.0 g of crude LBP was throughly dissolved in 100 mL of water and the solution was filter with a filter paper. Whereafter, alcohol (80% v/v) was added into the filtrate. After stored at 4℃ overnight，LBP was collected by centrifugation (5000 rpm, 5 min) and freeze-dried.

1.2 Preparation of Aminated reduced graphene oxide (NH_2-rGO)

200 mg of graphene oxide powder was dispersed into 200 mL of water using the ultrasonics processor. 600 μl of ammonium hydroxide and 2.4 mL of ethylenediamine were then added into the graphene oxide solution. The mixture was centrifuged (8000 rpm, 10 min) to collect the product after refluxed at 95°C for 6 h with stirring. Eventually, the product was diluted and mixed with 50 μl of hydrochloric acid (36% w/w).

1.3 Decoloration of purified LBP

0.8 g of purified LBP was dissolved in 15 mL of water and mixed with 4 mL of prepared NH_2-rGO. After decolored for 20 min, the mixture was centrifuged (8000 rpm, 10 min) to collect the supernatant. This procedure was repeated five times and supernatant was filtered to remove the residual NH_2-rGO.

2. Characterization of LBP-iron (III) chelate (LBPIC)

2.2 Physical property and SEM of LBPIC

Different concentrations of LBPIC solution (0.25 mg/mL, 0.5 mg/mL, 1.0 mg/mL) were prepared and one of them (1.0 mg/mL) was stored at room

temperature for seven days to investigate the physical property and stability of LBPIC solution. Scanning electron microscope (SEM; S-4800 l Hitachi, Tokyo, Japan) was used to study the surface morphology of LBPIC.

2.3 FT-IR spectroscopy

The KBr pellet method was used to analysis the FT-IR spectrum of LBPIC and purified LBP ranging from 400 cm^{-1} to 4000 cm^{-1}. The purified LBP and LBPIC were mixed with KBr respectively, and the mixtures were pressed to form pellets which were measured on a Vetex70 FT-IR spectrophotometer (Bruker, Karlsruhe, Germany).

2.4 XRD and XPS

An X-ray diffractometer ((D8 Advance; Bruker, Germany) was used to record the X-ray diffraction patterns of LBPIC. The X-ray photoelectron spectroscopy (XPS) was carried out by an Axis Ultra DLD instrument (Kratos Analytical, Manchester, UK) which was equipped with an Al Kα X-ray source (1486.6 eV).

3. Optimization for the test conditions of peroxidase-like activity of LBP-iron (III) chelate

In order to evaluate the peroxidase-like activity of LBPIC under different pH and temperature, the reaction system consists of acetate buffer (0.01 M), H_2O_2 (25 mM), TMB (1 mM) and LBPIC (25 μg/mL). Acetate buffer solutions whose pH value ranging from 2.0 to 7.0 was used to test effect of pH at room temperature and the reaction time was 90 s. The reaction condition was set

from 20 to 70℃ to investigate the temperature effect on enzyme activity of LBPIC, and each group reacted for 180 s. After completion of reactions, a microplate reader (Multiskan MK3, Thermo Fisher Scientific, US) was used to measure the absorbances at 652 nm of all groups. Additionally, to explore the effect of the concentration of LBPIC and reaction time on the TMB/H_2O_2 system, a series of experimental group containing disparate concentration of LBPIC (0-45 μg/mL) was set and the absorbances at 652 nm of each group was recorded every 60 s.

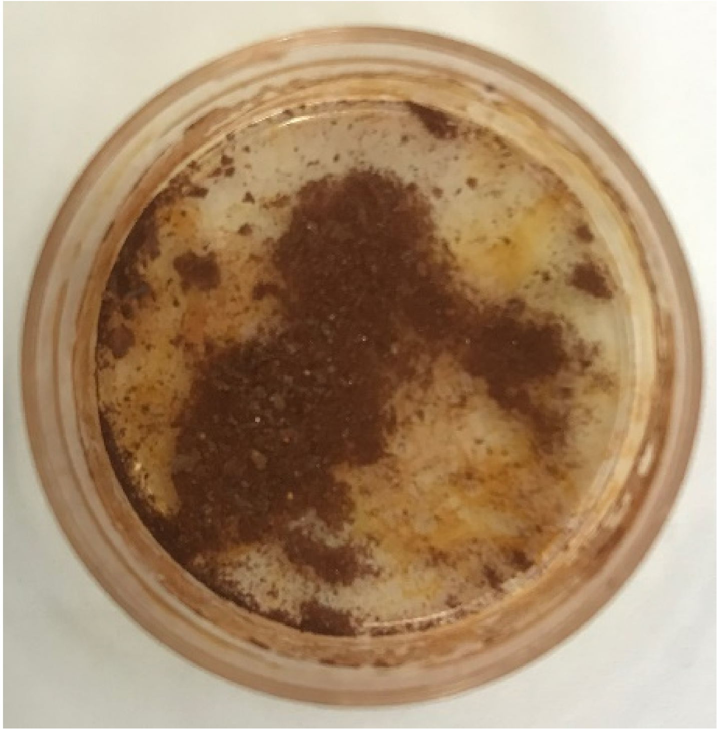

Figure S1. LBPIC (*Lycium barbarum* polysaccharide-iron (III) chelate) powder

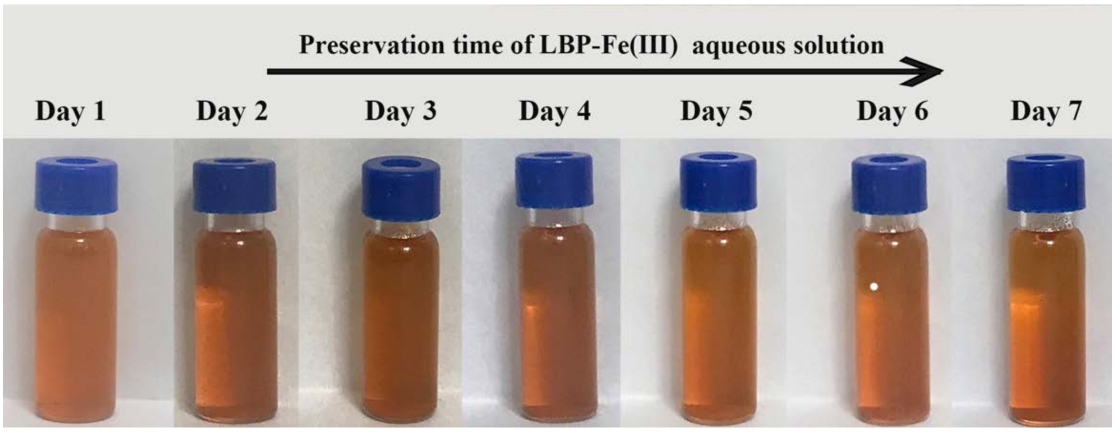

Figure S2. Storage stability test of LBPIC in water solution (1.0 mg/mL) for a week

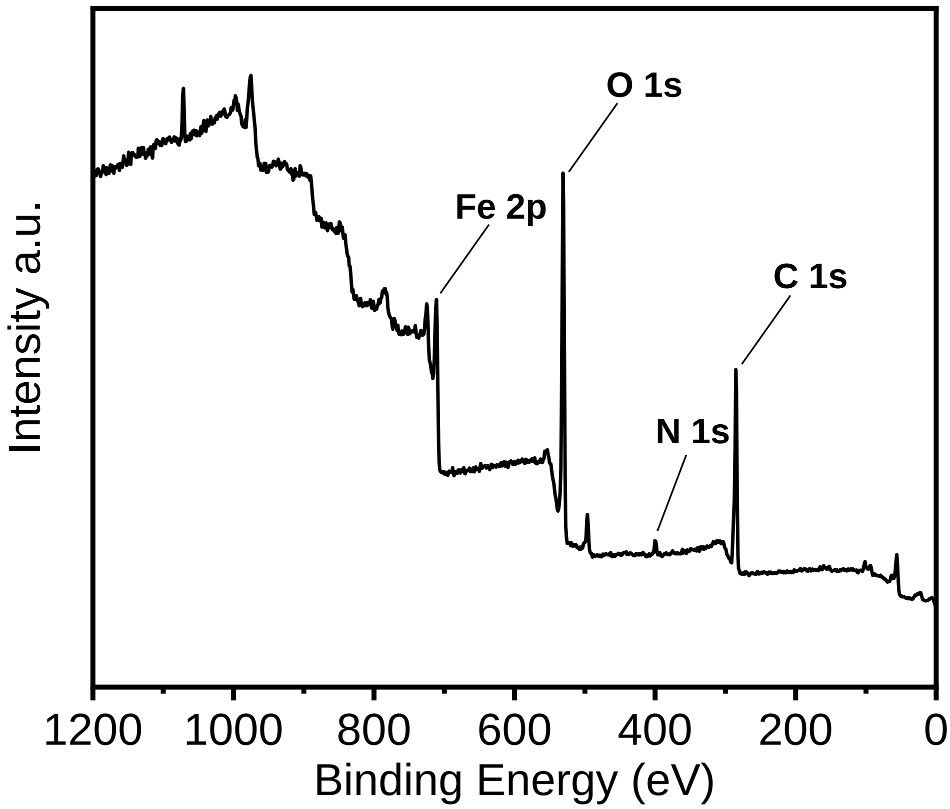

Figure S3. XPS spectrum of LBPIC

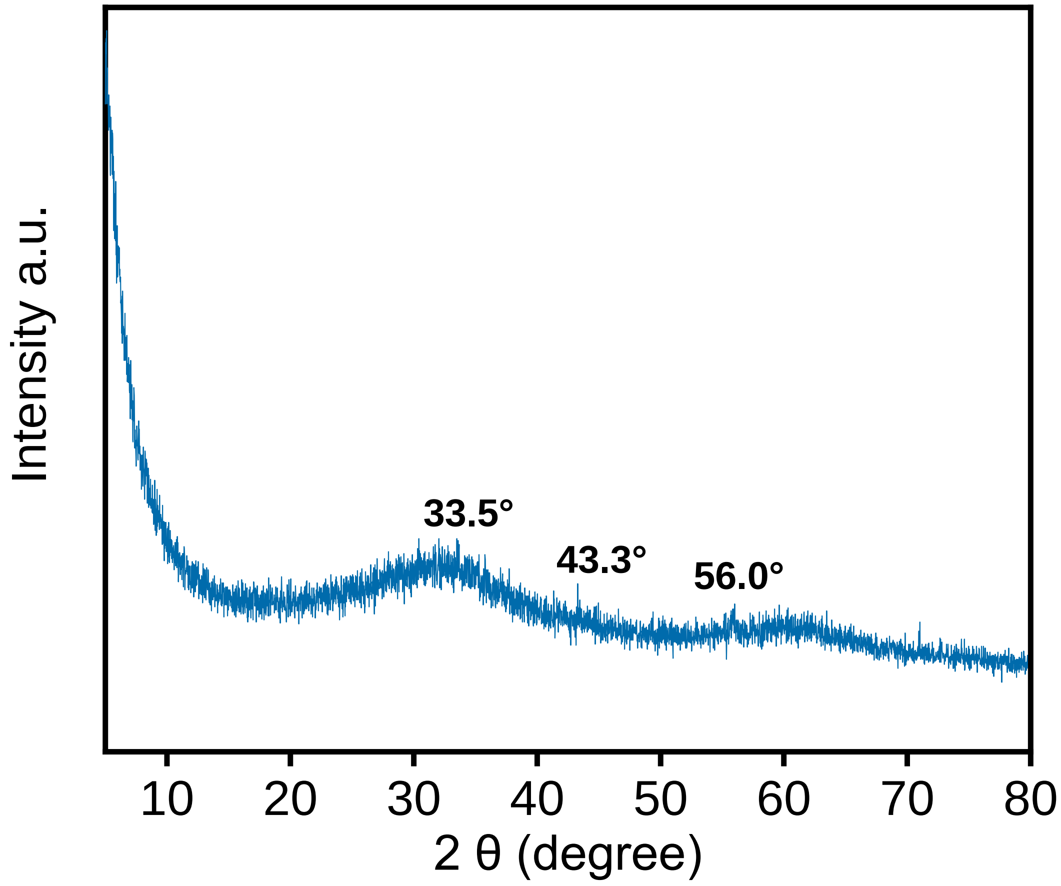

Figure S4. XRD spectrum of LBPIC

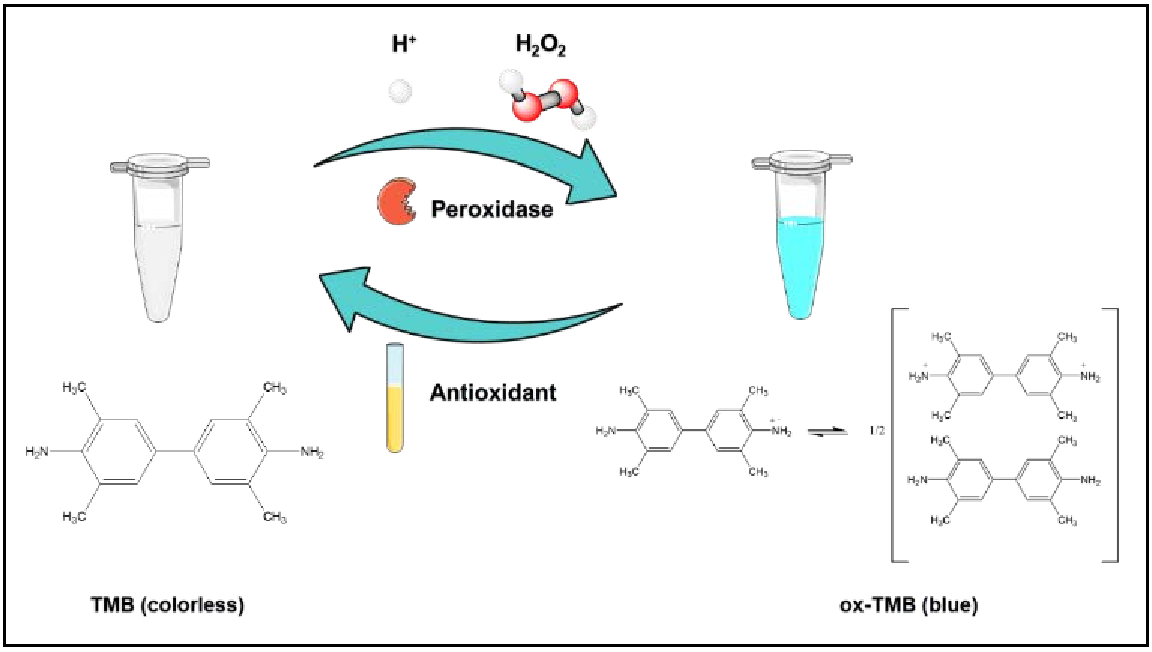

Figure S5. The color change mechanism of TMB/H_2O_2 system

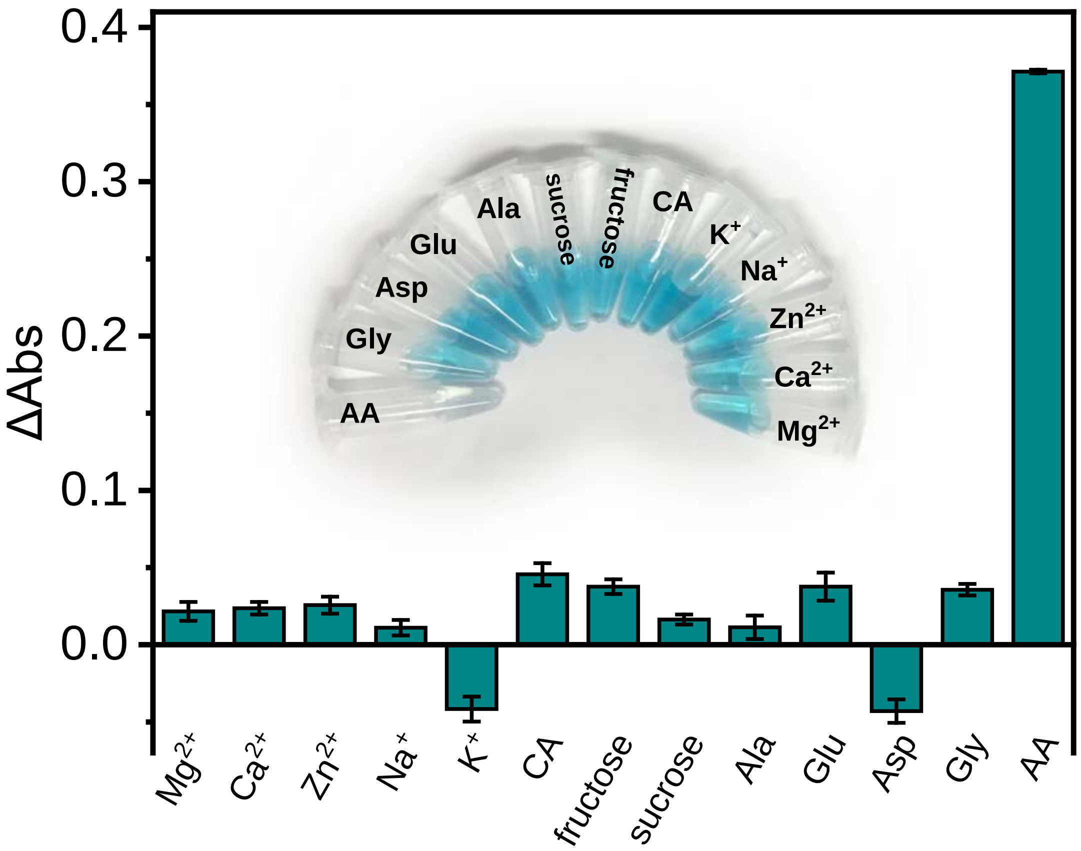

Figure S6. Selectivity analysis of AA over some common interfering substances coexisting in commercial compound fruit and vegetable beverages.

Table S1 The kinetic parameters of LBP-iron (III) chelate and some other peroxidase mimetics with TMB and H_2O_2 as substrates.

Catalyst	K_m（mM）		V_{max}（10^{-8} mM·s^{-1}）		Reference
	TMB	H_2O_2	TMB	H_2O_2	
HRP	0.43	3.70	10.00	8.71	[1]
IrO2/GO	0.56	5.19	32.80	20.80	[2]
3CoV-400	0.18	0.12	57.8	5.3	[3]
PBMNPs3	0.31	323.60	1.06×10^5	1.17×10^5	[4]
N-SCSs	0.15	81.53	2.21×10^4	2.33×10^4	[5]
LBP-iron (III) chelate	0.16	5.54	32.21	35.79	This work

K_m: Michaelis-Menten constant, V_{max}: maximal reaction velocity

Table S2 Comparison of different materials for the detection of AA.

material	Linear range (μM)	LOD (μM)	Incubation time(min)	Reference
Pt/CeO2	0.5-30	0.08	10	[6]
Dex-FeMnzyme	1-30	1.17	15	[7]
SNC	100-5000	80	5	[8]
CP600-6	8-80	8	15	[9]
LBP-iron (III) chelate	2-100	1.51	3	This work

LOD: limit of detection

References

1. Lu, Y.; Ye, W.C.; Yang, Q.; Yu, J.; Wang, Q.; Zhou, P.P.; Wang, C.M.; Xue, D.S.; Zhao, S.Q. Three-dimensional hierarchical porous PtCu dendrites: A highly efficient peroxidase nanozyme for colorimetric detection of H_2O_2. *Sens. Actuators, B* **2016**, 230, 721-730, doi:10.1016/j.snb.2016.02.130.
2. Sun, H.Y.; Liu, X.L.; Wang, X.H.; Han, Q.S.; Qi, C.; Li, Y.M.; Wang, C.; Chen, Y.X.; Yang, R. Colorimetric determination of ascorbic acid using a polyallylamine-stabilized IrO_2/graphene oxide nanozyme as a peroxidase mimic. *Microchim. Acta* **2020**, 187, doi:ARTN 11010.1007/s00604-019-3897-4.
3. Wang, Y.; Chen, C.; Zhang, D.; Wang, J. Bifunctionalized novel Co-V MMO nanowires: Intrinsic oxidase and peroxidase like catalytic activities for antibacterial application. *Appl. Catal., B* **2020**, 261, 118256, doi:ARTN 11825610.1016/j.apcatb.2019.118256.
4. Zhang, X.Q.; Gong, S.W.; Zhang, Y.; Yang, T.; Wang, C.Y.; Gu, N. Prussian blue modified iron oxide magnetic nanoparticles and their high peroxidase-like activity. *J. Mater. Chem.* **2010**, 20, 5110-5116, doi:10.1039/c0jm00174k.
5. Xi, J.Q.; Wei, G.; Wu, Q.W.; Xu, Z.L.; Liu, Y.W.; Han, J.; Fan, L.; Gao, L.Z. Light-enhanced sponge-like carbon nanozyme used for synergetic antibacterial therapy. *Biomater. Sci.* **2019**, 7, 4131-4141, doi:10.1039/c9bm00705a.
6. Liu, X.L.; Wang, X.H.; Qi, C.; Han, Q.S.; Xiao, W.; Cai, S.F.; Wang, C.; Yang, R. Sensitive colorimetric detection of ascorbic acid using Pt/CeO_2 nanocomposites as peroxidase mimics. *Appl. Surf. Sci.* **2019**, 479, 532-539, doi:10.1016/j.apsusc.2019.02.135.
7. Han, X.M.; Liu, L.; Gong, H.Y.; Luo, L.P.; Han, Y.R.; Fan, J.W.; Xu, C.F.; Yue, T.L.; Wang, J.L.; Zhang, W.T. Dextran-stabilized Fe-Mn bimetallic oxidase-like nanozyme for total antioxidant capacity assay of fruit and vegetable food. *Food Chem.* **2022**, 371, 131115, doi:ARTN 13111510.1016/j.foodchem.2021.131115.
8. Chen, Y.; Jiao, L.; Yan, H.; Xu, W.; Wu, Y.; Wang, H.; Gu, W.; Zhu, C. Hierarchically porous S/N codoped carbon nanozymes with enhanced peroxidase-like activity for total antioxidant capacity biosensing. *Anal. Chem.* **2020**, 92, 13518-13524, doi:10.1021/acs.analchem.0c02982.
9. Lou, Z.P.; Zhao, S.; Wang, Q.; Wei, H. N-Doped carbon as peroxidase-like nanozymes for total antioxidant capacity assay. *Anal. chem.* **2019**, 91, 15267-15274, doi:10.1021/acs.analchem.9b04333.
